# Differentiation of physical and chemical cross-linking in gelatin methacryloyl hydrogels

**DOI:** 10.1038/s41598-021-82393-z

**Published:** 2021-02-05

**Authors:** Lisa Rebers, Raffael Reichsöllner, Sophia Regett, Günter E. M. Tovar, Kirsten Borchers, Stefan Baudis, Alexander Southan

**Affiliations:** 1 Institute of Interfacial Process Engineering and Plasma Technology, University of Stuttgart, Stuttgart, Germany; 2 Christian Doppler Laboratory for Advanced Polymers for Biomaterials and 3D Printing, Institute of Applied Synthetic Chemistry, TU Wien, Vienna, Austria; 3 Fraunhofer Institute for Interfacial Engineering and Biotechnology, Stuttgart, Germany

**Keywords:** Bioinspired materials, Biomaterials - proteins, Biomaterials, Gels and hydrogels, Polymers, Rheology, Mechanical properties, Gels and hydrogels, Polymers, Rheology

## Abstract

Gelatin methacryloyl (GM) hydrogels have been investigated for almost 20 years, especially for biomedical applications. Recently, strengthening effects of a sequential cross-linking procedure, whereby GM hydrogel precursor solutions are cooled before chemical cross-linking, were reported. It was hypothesized that physical and enhanced chemical cross-linking of the GM hydrogels contribute to the observed strengthening effects. However, a detailed investigation is missing so far. In this contribution, we aimed to reveal the impact of physical and chemical cross-linking on strengthening of sequentially cross-linked GM and gelatin methacryloyl acetyl (GMA) hydrogels. We investigated physical and chemical cross-linking of three different GM(A) derivatives (GM10, GM2A8 and GM2), which provided systematically varied ratios of side-group modifications. GM10 contained the highest methacryloylation degree (DM), reducing its ability to cross-link physically. GM2 had the lowest DM and showed physical cross-linking. The total modification degree, determining the physical cross-linking ability, of GM2A8 was comparable to that of GM10, but the chemical cross-linking ability was comparable to GM2. At first, we measured the double bond conversion (*DBC*) kinetics during chemical GM(A) cross-linking quantitatively in real-time via near infrared spectroscopy-photorheology and showed that the *DBC* decreased due to sequential cross-linking. Furthermore, results of circular dichroism spectroscopy and differential scanning calorimetry indicated gelation and conformation changes, which increased storage moduli of all GM(A) hydrogels due to sequential cross-linking. The data suggested that the total cross-link density determines hydrogel stiffness, regardless of the physical or chemical nature of the cross-links.

## Introduction

Gelatin methacryloyl (GM, GelMA) hydrogels are considered as promising biomaterials. They are commonly investigated for biomedical applications such as scaffolds for tissue engineering or drug delivery^1,2^. Properties of GM solutions, e.g. viscosity and temperature of physical gelation, as well as characteristics of the cross-linked hydrogels, e.g. water content and mechanical properties, can be tuned by rather simple experimental adjustments: commonly accepted methods include varying the GM concentration and/or the degree of methacryloylation (DM) before cross-linking^3–5^, adjusting the irradiation dose used during photo-cross-linking, or combining other (photo-cross-linkable) materials with GM^2^. These approaches focus mainly on the ability of GM to form chemical cross-links and neglect the physical interactions of gelatin molecules, which are less pronounced after methacryloylation than in unmodified gelatin, but can still be sufficient to form physical cross-links and gels, respectively.

Several studies investigated in how far the formation of both, physical and chemical cross-links, in GM hydrogels can act synergistically to improve mechanical properties. To this end, a sequential cross-linking procedure of GM hydrogels was applied by several authors, which increased the maximum strength^6^, compressive modulus^7,8^, storage modulus^9,10^ and E-modulus distinctly^11^. During this sequential cross-linking procedure, the hydrogel precursor solutions were held at rather low temperatures, e.g*.* 4 °C, and were chemically cross-linked afterwards. The observed strengthening effect was usually not temperature-dependent after chemical cross-linking^6–9^—in contrast to gelatin hydrogels chemically cross-linked with bis(vinylsulfonyl)methane^12,13^. However, a recent study suggests that the former must not always be the case^14^. The impact of the sequential cross-linking procedure on the mechanical properties is plausible because it increases the total cross-link density in GM-hydrogels^6–9,14^.

It must be assumed that the underlying effects differ depending on the degree of modification of the GM. In GMs with a low DM, approx. 0.3 mmol g^−1^, complete modification of accessible amino groups of the raw material occurs, but little to no modification of hydroxyl groups (e.g. GM2^3,15^). Such derivatives form physical hydrogels above 10 °C upon cooling similar to unmodified gelatin^3^. This indicates that effective physical cross-linking can take place, presumably by the formation of triple helices, in spite of the methacryloylation^6–9,14^.

In contrast to this, solutions prepared from GMs with much higher DMs, e.g. 0.8 mmol g^−1^ ^6^ or 0.99 mmol g^−1^ ^9^, are unable to form obvious hydrogels at temperatures above 10 °C^6,9^. Therefore, it can be hypothesized that physical cross-linking is largely suppressed by the modification of amino and hydroxyl groups. In this case, the strengthening effect might be due to a more efficient chemical cross-linking of methacryloyl functions due to closer packaging of GM coils^8,9^ or the formation of hydrophobic methacryloyl domains induced by the initial cooling step^6^. Thus, the chemical cross-linking would result in a higher double bound conversion (*DBC*) while cross-linking of physical gels would rather reduce the *DBC* comparatively*.*

Rizwan et al*.* did Fourier transform infrared spectroscopy with solely chemically cross-linked and sequentially cross-linked GM hydrogels and found first qualitative indications of a higher *DBC* in sequentially cross-linked GM hydrogels^8^. Van Vlierberghe et al*.* introduced a method using high-resolution magic angle spinning (HR-MAS) NMR spectroscopy to investigate the final *DBC* and found *DBCs* lower than 40%^16^. Billiet et al*.* used the same method and implicated a non-linear relationship between qualitative methods, such as swelling measurements, and quantitative methods predicating cross-linking efficiencies^17^.

In this study, we aimed to shed further light into the participation of physical and chemical cross-links on the mechanical properties of GM hydrogels prepared either by the sequential cross-linking procedure or the classical cross-linking procedure omitting the initial cooling step. Therefore, we used three different GM derivatives for hydrogel preparation, which differed in their ability to form either chemical or physical cross-links: (1) GM2, a GM derivative with a relatively low DM of 0.29 mmol g^−1^, thus with a low density of chemically cross-linkable groups, but a high physical cross-linking potential. (2) GM2A8, a methacryloylated and acetylated gelatin (GMA) derivative with a DM of 0.36 mmol g^−1^ which was comparable to GM2, but with the low physical cross-linking potential due to the high total degree of modification. (3) GM10, a GM derivative with a DM of 0.95 mmol g^−1^, thus also having a low tendency to form physical cross-links but with a high density of chemically cross-linkable groups.

By real time-near infrared spectroscopy combined with photorheology, we aimed to measure the *DBC* and gelation kinetics during cooling and during the photo-induced cross-linking reaction of methacryloyl functions of GM in situ for the first time. Both cross-linking methods, either the classical method at 37 °C or sequential cross-linking including an initial cooling step, were in the focus of this investigation. Additionally, the formation of hierarchical structures such as triple helices at cross-linking conditions were investigated qualitatively by circular dichroism spectroscopy and quantitatively by differential scanning calorimetry.

Putting the data on chemical and physical cross-linking into context, we hope to contribute to understanding the processes underlying the formation of GM hydrogels.

## Methods

### Materials

Acetic anhydride (AcAnh), Dulbecco’s phosphate buffered saline with MgCl_2_ and CaCl_2_ (PBS), methacrylic anhydride (MAAnh) as well as sodium hydroxide (NaOH) were purchased from Sigma Aldrich (Darmstadt, Germany). Sodium 3-trimethylsilyl-propionate-2,2,3,3-d4 (TMSP) was bought from Merck (Darmstadt, Germany). Other reagents were purchased from the following sources (given in parentheses): deuterium oxide (D_2_O, Deutero; Kastellaun, Germany for ^1^H-NMR measurements, Eurisotop; Saarbrücken, Germany for NIR-Photorheology), Gelatin (type B, limed, bovine bone, 232 Bloom, standard viscosity = 4.5 mPa s; Gelita; Eberbach, Germany). Dialysis membranes (MWCO 12 kDa–14 kDa) were purchased from Medicell International Ltd. (London, UK). The photoinitiator lithium phenyl-2,4,6-trimethylbenzoylphosphinate (LAP) was synthesized according to Fairbanks et al.^18^. The ^1^H-NMR spectrum (500 MHz, D_2_O) was in accordance with the literature, i.e., 7.74 (m, 2H), 7.56 (m, 1H), 7.46 (m, 2H), 6.87 (s, 2H), 2.22 (s, 3H), 2.04 (s, 6H).

### Gelatin methacryloyl (acetyl) synthesis procedure

Gelatin methacryloyl (GM) and gelatin methacryloyl acetyl (GMA) derivatives GM2, GM10, and GM2A8 were prepared as described previously^15^. Briefly, gelatin (25.01 g) was dissolved in deionized water (250 mL) at 37 °C and the solution pH was adjusted to 7.3 using an automatic titration device. Within 30 min, 2.70 g or 13.49 g of MAAnh were added, respectively, for GM2 or GM10, which corresponds to a two-fold or ten-fold molar excess of MAAnh relative to the amino groups present in gelatin (0.35 mmol g^−1^)^5^. For GM2A8 synthesis, 7.15 g of acetic anhydride (AcAnh) were added dropwise after 2 h of methacryloylation reaction within 30 min, resulting in an eight-fold AcAnh molar excess relative to the amino-groups present in gelatin^5^. In all cases, the reaction mixture was stirred vigorously for 5 h in total, keeping its pH constantly between 7.0 and 7.4. The reaction mixture was filtrated subsequently and its pH was adjusted to 9.5. After 2 days storage at 4 °C, the solution was dialyzed for 4 d against deionized water, the first 3 d at room temperature and the last day at 40 °C. After that, the solution pH was adjusted to 8.5 and freeze-dried.

The degree of methacryloylation (DM) was determined using the TMSP-method as described by Claaßen et al*.*^15^ utilizing ^1^H-NMR spectroscopy. The used GM-batches had the following DMs: 0.29 mmol g^−1^ (GM2), 0.95 mmol g^−1^ (GM10) and 0.36 mmol g^−1^ (GM2A8). The degree of acetylation of GM2A8 was reported before as 0.76 ± 0.03 mmol g^−1^ ^3^. Thus, the total modification degree of GM2A8 was assumed to be ~ 1.12 mmol g^−1^. The ^1^H-NMR spectra of GM(A) derivatives are given in the Supporting Information (Figure S1).

### Circular dichroism (CD) spectroscopy

For CD spectroscopy, 0.1% (w/w) gelatin or gelatin derivative solutions were prepared as follows: 2 mg gelatin or gelatin derivative were dissolved in 1998 mg PBS over night at 37 °C. The solutions were kept at 37 °C until they were filled into the quartz glass measuring cuvette (cell length 0.1 mm) and transferred directly into the 37 °C pre-warmed CD spectrometer (Jasco J-815, Pfungstadt, Germany). Firstly, CD spectra at 37 °C were recorded: 50 spectra accumulations were collected between 200 and 260 nm, whereby the temperature was kept constant (± 0.1 °C).

Afterwards, the gelatin or gelatin derivative solutions were removed from the CD spectrometer, stored at 21 °C and the temperature was maintained for 20 min, followed by cooling to 4 °C and maintaining the temperature for 40 min. Subsequently, CD-spectra were collected in the 4 °C pre-cooled CD spectrometer as described above. According to Martin and Schilstra^19^ the mean of CD between 250 and 260 nm was subtracted from CD spectra to correct the vertical drift. CD spectra of PBS were recorded as baseline at 4 °C and 37 °C and subtracted from the gelatin and gelatin derivative CD spectra to obtain pure sample CD spectra. Three independent measurements of each solution were done.

Measured values are expressed as mean residue ellipticity *Ɵ*. For calculation of *Ɵ*, a mean molar mass per amino acid residue of 90.68 g mol^−1^ was used, based on the amino acid composition of the unmodified gelatin published by Sewald et al*.*^3^. For gelatin derivatives, we corrected the mean molar mass per amino acid residue due to inserted functional groups with DMs and degree of acetylation determined in this study^3^: 92.75 g mol^−1^ (GM2), 94.91 g mol^−1^ (GM2A8) and 97.46 g mol^−1^ (GM10).

### Differential scanning calorimetry (DSC)

For preparation of quantitative DSC measurements, a temperature and enthalpy calibration of the differential scanning calorimeter (Netzsch DSC 200 F3 Maia, Selb, Germany) was done as described by the manufacturer with hermetically sealed high purity standards of bismuth, indium, zinc, tin, caesium chloride and rubidium nitrate with a temperature rate of 5 °C s^−1^.

Gelatin or gelatin derivative solutions in PBS were prepared as follows: 100 mg gelatin or gelatin derivative was dissolved in 800 mg PBS at 37 °C. A defined amount of gelatin or gelatin derivative solution was weighed into a crucible, which was subsequently hermetically sealed. Afterwards, DSC measurements were performed in three cycles from − 10 °C to 80 °C at 5 °C s^−1^ with three independent samples for gelatin and each gelatin derivative. The renaturation level (*X*_*DSC*_) determined by DSC was calculated according to Bigi et al*.*^20^ utilizing Eq. (1), whereby ${\Delta H}_{m}$ was the denaturation enthalpy of the sample and $\Delta {H}_{G,m}$ the denaturation enthalpy of the unmodified gelatin.

1 $${X}_{DSC}= {\Delta H}_{m}/{\Delta H}_{G,m}$$

### Real time-near infrared spectroscopy-photorheology

Real time (RT)-near infrared spectroscopy (NIR)-photorheology is a hyphenated technique of a rheological measurement during in situ photo-cross-linking of a formulation, coupled with RT-NIR spectroscopy in reflection mode of the respective sample. The setup consists of an Anton Paar (Graz, Austria) MCR302 WESP rheometer, a Bruker (Billerica, USA) Vertex 80 IR spectrometer and an Excelitas (Waltham, USA) Omnicure S2000 UV source (320–500 nm), as described in Gorsche et al*.*^21^. The rheometer had a glass base plate with a covering hood (both with a Peltier element) and was used with a PP08 tool (plate-plate geometry, 8 mm plate diameter, aluminium).

For sample preparation, an LAP stock solution was prepared by dissolving 10 mg of LAP in 990 µL of D_2_O. GM2, GM10 or GM2A8 hydrogel precursor solutions were prepared by dissolving 100 mg of the respective gelatin derivative in 780 µL of D_2_O and 20 µL of LAP stock solution. A solution of unmodified gelatin was prepared in the same way. Hydrogel precursor solutions were pre-warmed to 37 °C before RT-NIR-photorheology measurements for decreased viscosity and prevention of premature physical gelation. Each GM hydrogel precursor solution was measured with two different temperature settings. During these temperature settings the storage modulus (*G′*) of gelatin (derivative) solutions was measured to investigate the physical cross-linking. Firstly, the hydrogel precursor solutions were cross-linked following the classical method and the double bond conversion (*DBC*) was monitored at constant 37 °C. Secondly, a temperature profile (see Figure S2 in the Supporting Information) was investigated, representing the sequential cross-linking method. The measurements at 37 °C were performed in quadruples and the sequential cross-linking method was performed in triplicates. The sequential cross-linking method contained three temperature levels: during the first minute of the measurement, the temperature was held at 37 °C. Then, the temperature was decreased to 21 °C and held at 21 °C for the following 20 min. Afterwards, the temperature was further decreased to 4 °C and held at 4 °C for 40 min. From this point on, the *DBC* monitoring and chemical cross-linking reaction for the temperature profile were started. Figure S2 in the Supporting Information precisely illustrates the time points and durations of cooling steps, the NIR-data collection, and the chemical cross-linking, respectively.

Rheological data were measured throughout the experiments in oscillation mode with a constant frequency of 1 s^−1^, a constant strain of 1%, a gap size of 1 mm, and a gap filling of 53 µL. The measuring gap was filled in the measuring position and water evaporation was prevented with a surrounding paraffin ring around the sample cylinder. Rheological data was recorded with a frequency of 5 data point s^−1^ before UV cross-linking and 1 data point s^−1^ during UV cross-linking with the help of the software RHEOPLUS/32 V3.62.

For chemical cross-linking, UV light was guided from a UV source via a bifurcated light guide through the glass base plate of the rheology setup. The UV intensity was measured at the surface of the glass plate with a spectrophotometer (Ocean Optics USB2000+, Ostfildern, Germany) and was adjusted to 10 mW cm^−2^. Samples were cured for 5 min to ensure a steady state of chemical cross-linking.

NIR spectra were taken in transmission mode with a spectral resolution of 4 cm^−1^, leading to a time resolution (scanning speed) of about 2 spectra s^−1^. Contrary to the published RT-NIR-photorheology method^21^, background measurements were taken with a gelatin solution, which was prepared as stated above. NIR spectra data acquisition was started 5 s prior to initiation of the chemical cross-linking by UV irradiation for later calculation of methacryloyl signal integrals before irradiation ($Integral \left(t=0\right)$)*.* NIR spectra were recorded with the software OPUS 7.0. Method “F” was used for integration of methacryloyl signals, which determines the local baseline at the relevant spectral range. Furthermore, this method was adjusted for each of the principal measurements and was the same for measurement repetitions. The methacryloyl C=C double bond signal was integrated in the wavenumber range of 6220–6110 cm^−1^.

The *DBC* was calculated in % from the integrals with the following Eq. (2):

2 $$DBC \left[\%\right]=(1-\frac{Integral \left(t\right)}{Integral \left(t=0\right)})\cdot 100$$

*Integral(t* = *0)* was determined via averaging of integrals of a 5 s window before the initiation of the chemical cross-linking reaction. The final *DBC* was determined via averaging of calculated *DBCs* over the last 10 s of the measurement.

### Statistical analysis

The results are presented as mean values of at least three independently performed measurements with the respective standard deviation. Statistical significances were determined by a one-tailed ANOVA. Significant differences were defined for *p* values lower than *α* = 0.05, *α* = 0.01 and *α* = 0.001.

## Results and discussion

### Physical interactions in modified gelatin derivatives

**Figure 1:**
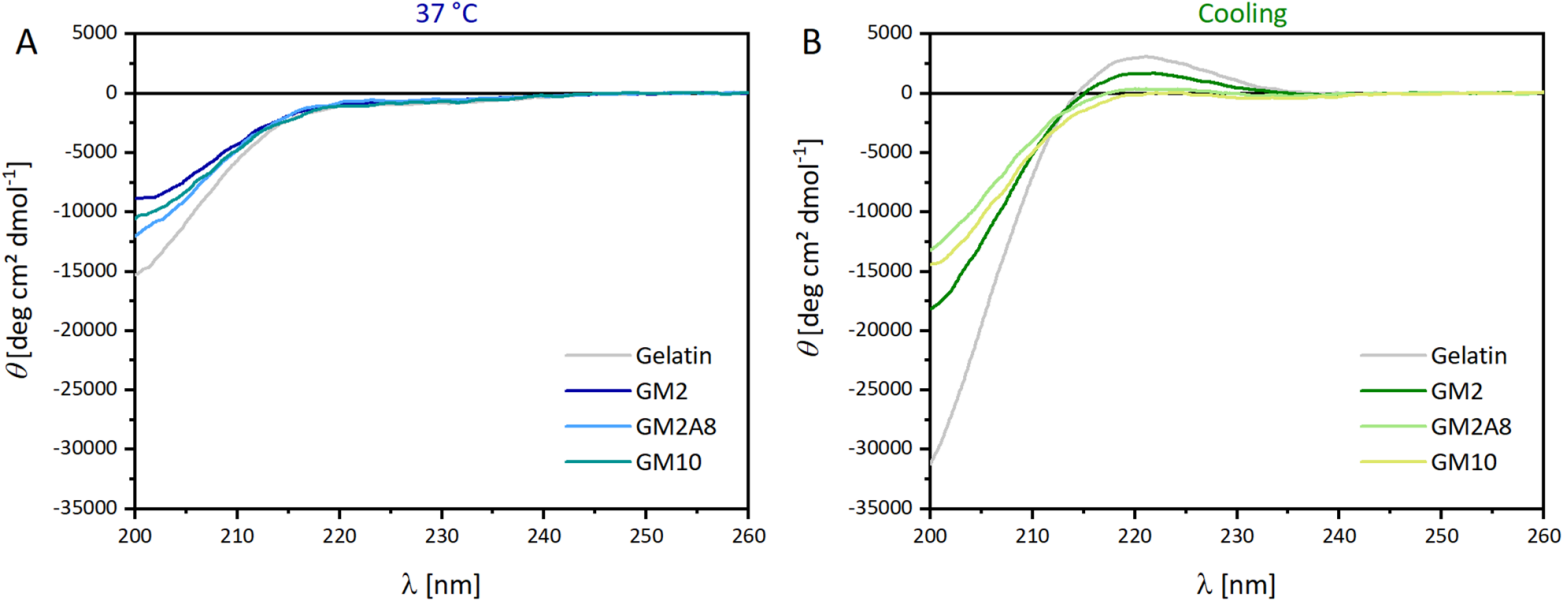
Molar ellipticity *Ɵ* of unmodified gelatin, GM2, GM2A8, and GM10. The spectra were recorded at 37 °C (**A**) or after the temperature protocol, whereby the 37 °C warm solution was cooled to 21 °C for 20 min followed by cooling to 4 °C for 40 min (**B**, cooling). For gelatin and GM2 a clear positive signal around 220 nm was detected after cooling, which suggested the presence of alpha helices. The signal was absent for all solutions measured at 37 °C, and the negative molar ellipticity of all solutions around 200 nm emphasized the presence of random coil structures. The figure was created with Origin 2019b (https://www.originlab.com/2019b).

**Figure 2:**
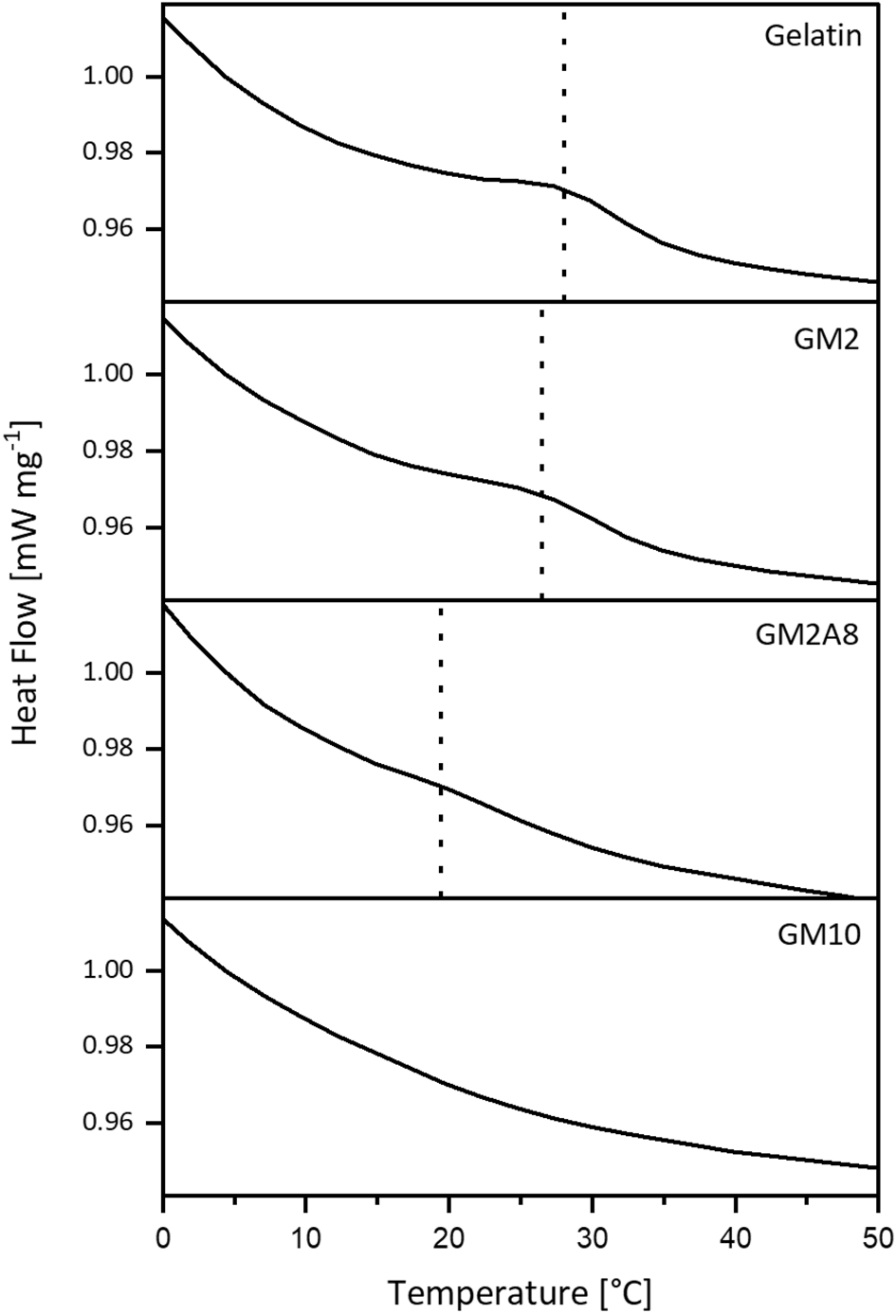
Differential scanning calorimetry (DSC) curves of unmodified gelatin, GM2, GM2A8, and GM10. Vertical dotted lines indicate the melting temperatures. The figure was created with Origin 2019b (https://www.originlab.com/2019b).

We first aimed at a detailed characterization of the state that is obtained after the initial cooling step in a sequential cross-linking procedure, before taking a closer look at the chemical cross-linking process of the studied gelatin derivatives. For this purpose, we used CD spectroscopy and DSC to investigate conformations of gelatin (derivatives) in aqueous solution. In this context, the ability of GM(A) derivatives to form typical gelatin triple helices as well as unordered polypeptide chains, at 37 °C and after cooling procedure, is of special interest. CD spectroscopy is highly sensitive towards conformation changes and thus gives some information about specific protein conformations. DSC was used to quantify physical interactions by measuring the enthalpy change upon heating. CD spectra are given in Fig. 1 and Figure S3 in the Supporting Information, DSC curves are shown in Fig. 2.

The CD spectra of all solutions recorded at 37 °C were negative from 200 nm to roughly 250 nm. The gelatin and GM(A) solutions showed a negative CD band around 200 nm, which was more pronounced for gelatin than for GM(A). Apart from that, the spectra at 37 °C had no distinct features. The CD spectra of all gelatin (derivative) solutions changed due to the cooling procedure, indicating strong conformation changes: the negative band around 200 nm was more pronounced for all solutions compared to the spectra recorded at 37 °C. Furthermore, a clear positive CD band around 221 nm was observed for gelatin (3070.3 deg cm^2^ dmol^−1^), GM2 (1629.5 deg cm^2^ dmol^−1^) and GM2A8 (344.8 deg cm^2^ dmol^−1^). For GM10, a slightly negative CD value was observed (− 57.2 deg cm^2^ dmol^−1^) at 221 nm. In addition, a negative band between 230 and 250 nm stood out for highly modified GM2A8 and GM10. A more detailed graphic of this band is shown in Figure S4 in the supporting information.

These temperature-dependent changes in CD spectra can be ascribed to temperature-sensitive conformation changes of gelatin (derivatives) in solution. A negative band in CD spectra of warm gelatin and collagen solutions around 200 nm was shown to be connected to random polypeptide chains^22^, whereby the positive band around 220 nm in combination with an intense negative band around 200 nm in cooled solutions is characteristic for triple helices^23–25^. The triple helix CD spectra resemble that of poly-proline II helices (*trans* form of poly-proline), implying that the three single left-handed helices of the triple helix are in the same conformation as *trans* poly-proline^26^. This conformation is solely stabilized in the triple helix structure^27^. That is why we inferred that all gelatin (derivative) solutions had a random polypeptide chain conformation at 37 °C. Furthermore, GM2 and GM2A8 solution showed pronounced triple helix conformations after cooling. This was in accordance with results of other studies investigating sequential GM hydrogels^8,28,29^ in which a 0.2 mg mL^−1^ solution in distilled water was cooled for 2 h at 4 °C^28,29^ or a 0.1% (w/v) solution was cooled for 1 h at 4 °C^8^.

In addition, the amount of triple helices after the cooling procedure decreased with increasing degree of modification (gelatin > GM2 > GM2A8 > GM10). These results were consistent with another CD study of GMs of Zhu et al*.* showing that an increasing DM increased the random polypeptide chain conformation and decreased the triple helix conformation^28^. Furthermore, Zhu et al*.* investigated GM with a DM of ~ 0.32 mmol g^−1^ (DS_100), which was very close to the GM2 we used in this study (DM = 0.29 mmol g^−1^). They detected just a very slight positive band of DS_100 at 222 nm, whereas we detected a clear positive band at 221 nm for GM2. This might be explained by experimental differences: our cooling period was shorter, our GM concentration five times higher and we used PBS instead of deionized water. It was shown before that all these differences impact triple helix renaturation^22,25^.

Negative bands at wavelengths > 230 nm with a maximum at ~ 238 nm of gelatin and collagen were previously correlated with a right-handed helical poly-proline I conformation^26^. This poly-proline I conformation of single chains contains *cis* peptide bonds and thereby is the *cis*-peptide form of poly-proline. The helical pitch of the right-handed poly-proline I helix is smaller (5.6 Å turn^−1^, 3.3 residues turn^−1^) than the pitch of the left-handed poly-proline II conformation (9.3 Å turn^−1^, 3.0 residues turn^−1^), making it more compact^30^. It was shown that the poly-proline I conformation stabilized single chains^31^ and is preferred in organic solvents^32^. Furthermore, a correlation with the positive band at ~ 220 nm was found: the smaller the positive band at ~ 220 nm, the bigger the negative band at ~ 238 nm. Thus, the destruction of triple helices into single helices due to high temperatures might be correlated with a *trans*–*cis*-transition of peptide bonds in single helices^26^. That is why we assigned the negative band at > 230 nm, which was measured for all GM(A) solutions, to single chains, which did not associate to triple helices during cooling. The molar ellipticity *Ɵ* at 238 nm after the cooling procedure was lowest for GM10 (− 319.9 deg cm^2^ dmol^−1^), followed by GM2A8 (− 203.1 deg cm^2^ dmol^−1^), GM2 (− 127.7 deg cm^2^ dmol^−1^) and gelatin (− 38.9 deg cm^2^ dmol^−1^). Thus, in GM10 the most single helices were built up and did not form critical nucleus structures to form triple helices, as proposed by Guo et al*.*^33^.

The CD spectroscopy findings were generally supported by the DSC measurements (Fig. 2). An endothermic peak was detected for all gelatin (derivative) solutions during heating except for GM10. The respective temperatures were interpreted as melting temperatures *T*_*m*_. The obtained *T*_*m*_, melting enthalpies *ΔH*_*m*_ and *X*_*DSC*_ are given in Table 1. *T*_*m,*_ *ΔH*_*m*_ as well as *X*_*DSC*_ generally decreased with increasing degree of modification up to the point that no values were obtained for GM10.

**Table 1:** Melting temperatures *T*_*m*_ and melting enthalpies *ΔH*_*m*_ obtained from the DSC curves shown in Fig. 2 as well as renaturation levels *X*_*DSC*_ calculated from DSC measurements.

	*T*_*m*_ (°C)	*ΔH*_*m*_ (J g^−1^)	*X*_*DSC*_ (%)
Gelatin	28.0 ± 0.7	0.69 ± 0.11	100
GM2	26.5 ± 0.7	0.46 ± 0.06	71.0
GM2A8	19.4 ± 1.0	0.03 ± 0.02	4.3
GM10	n.f	n.f	n.f

GM10 did not show any signal in the DSC measurements or the signal was too shallow to be analysed, so no values were found (n.f.). The measured melting enthalpies were significantly different from each other [p < 0.05 (gelatin, GM2), p < 0.001 (gelatin, GM2A8; GM2, GM2A8)].

This observation was in accordance with previous findings measured by temperature-dependent rheology for GM10, which showed no gelation or melting temperature between 10 and 40 °C^3^. However, in the previous experiments also for GM2A8 no physical gelation was detected. The results of GM2 were in accordance with previous DSC studies of GM derivatives with DMs of 89%^8^ and 97%^9^, where the percentage describes the relative amount of methacryloylated amino groups. In concert with the CD spectra, the endothermic peaks of the DSC curves for gelatin, GM2 and GM2A8 were assigned to triple helix denaturation^9,20,34^, thereby again indicating the existence of triple helices in all cooled solutions except for GM10 solutions.

The effect of methacryloylation on gelatin triple helix formation was discussed already in the early publication on GM by van den Bulcke et al*.*^5^. It was shown in different studies that the modification of gelatin impacts solution viscosities^3,4,35,36^ and melting temperature^3,5,35^, which was correlated with the helix content of the GM solutions. However, there was also evidence that there is not necessarily a correlation between melting temperature and helix content^24^. The impact of methacryloylation on triple helix renaturation was explained by reduced interchain or intrachain hydrogen bindings within the triple helices by chemical functionalization of amino and hydroxyl groups, on the one hand^28^. On the other hand, peptide sequences such as the glycine-proline-hydroxyproline tripeptide, which are known to participate in the triple helix formation^37^, are chemically modified especially in the highly modified derivatives, potentially hindering the (complete) formation of triple helices^28^.

The presented data strongly indicate conformational changes in all investigated GM(A) solutions due to cooling procedure: for all solutions a more pronounced band of random polypeptide chains was detected. Furthermore, for GM2 and GM2A8 triple helices were evidenced by CD spectroscopy and DSC measurements, but not for GM10. However, more single helices were detected in GM10 than in gelatin, GM2 and GM2A8 solutions.

### Physical gelation of gelatin and respective derivatives

**Figure 3:**
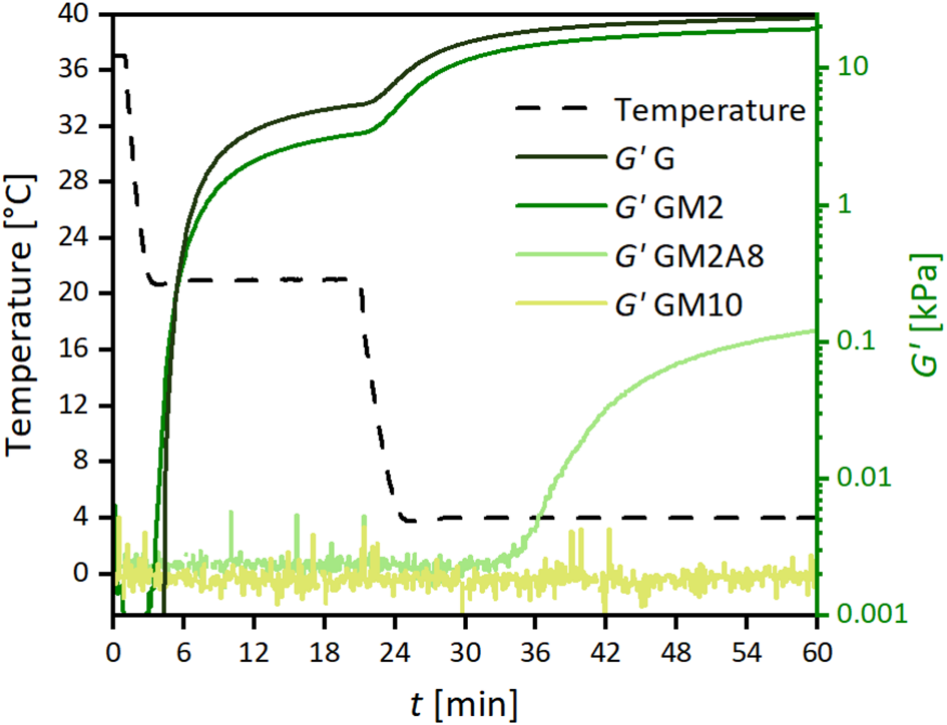
Evolution of storage modulus *G′* during the initial cooling protocol of the sequential cross-linking procedure for the tested gelatin derivatives and the raw material gelatin (G). Note that the data on the subsequently performed chemical cross-linking of the samples shown here is depicted in Fig. 4 and discussed in the next section. The figure was created with Origin 2019b (https://www.originlab.com/2019b).

The effect of the conformational changes induced by the cooling procedure during sequential cross-linking on the physical gelation ability of the gelatin raw material and its derivatives was analysed by measuring *G′* of the solutions by rheology, see Fig. 3.

Information about physical gelation speed *k*_*g,p*_ and the physical gelation delay time *t*_*d,p*_ were extracted and are collected in Table 2. The values were defined such that *k*_*g,p*_ is the slope of the initial linear part of the *G′(t)* curves (for unmodified gelatin and GM2 at 21 °C and 4 °C, for GM2A8 at 4 °C) and *t*_*d,p*_ is the intersection of the corresponding linear fit line with the baseline^21^. In accordance with the CD spectroscopy and DSC data discussed above and findings reported by Rebers et al*.*^6^ before, physical gelation was observed during sequential cross-linking (Table 2). Just gelatin and GM2 solutions formed physical gels during the period at 21 °C. This meets with the expectations based on the determined melting temperatures (Table 1), although the latter are commonly higher than the corresponding gelation temperatures.

**Table 2:** Parameters describing the physical gelation of gelatin derivative solutions using the sequential cross-linking protocol: physical gelation delay time *t*_*d,p*_, initial increase rate *k*_*g,p*_ of storage modulus *G′* upon cooling, and *G′* after cooling to 21 °C for 20 min and 4 °C for 40 min, respectively.

	Physical Gelation					
	21 °C			4 °C		
	*t*_*d,p*_ (s)	*k*_*g,p*_ (Pa s^−1^)	*G′* at end of period (kPa)	*t*_*d,p*_ (s)	*k*_*g,p*_ (Pa s^−1^)	*G′* at end of period (kPa)
Gelatin	251.1 ± 13.9	10.0 ± 1.0	5.40 ± 0.50	79.5 ± 1.8	24.8 ± 2.0	23.59 ± 1.93
GM2	226.7 ± 5.4	5 ± 0.1	3.33 ± 0.07	101.6 ± 0.6	21 ± 0.2	19.4 ± 0.01
GM2A8	–	–	–	981.0 ± 124.0	0.11 ± 0.01	0.124 ± 0.022
GM10	–	–	–	–	–	–

More specifically, delay times of the gelation events of GM2 and gelatin were in the same range. Unmodified gelatin gelled at 21 °C with a stiffness increase of 10.0 ± 1.0 Pa s^−1^ and the final *G′* after 20 min at 21 °C was 5.40 ± 0.50 kPa. Gelatin gelation at 4 °C resulted in a 2.5 times faster stiffness increase than for the 21 °C step and a *G′* of 23.59 ± 1.93 kPa. For GM2, the increase of *G′* at 21 °C was comparably slower (5.0 ± 0.1 Pa s^−1^), resulting in a *G′* of 3.33 ± 0.07 kPa when starting to cool to 4 °C. After cooling was complete, the increase of *G′* was accelerated so that for GM2, a final *G′* of 19.4 ± 0.01 kPa was reached at the end of the cooling protocol. In contrast to GM2, physical GM2A8 gels were formed only at 4 °C with a rather slow increase of *G′*, resulting in a low final value of 0.124 ± 0.02 kPa and GM10 solutions did not show any measurable response to cooling.

The results from physical gelation experiments of gelatin and GM(A) derivatives confirmed a tight correlation of gelation behaviour and degree of modification. While gelatin and GM2 formed gels even at 21 °C, GM2A8 only forms gels when cooled to 4 °C and GM10 did not gel even at 4 °C. Furthermore, this correlation could be qualitatively expanded towards the mechanical properties of physically cross-linked hydrogels as final storage moduli of gels after the 4 °C treatment decreased (gelatin > GM2 > GM2A8, no gels for GM10) with increasing degree of modification. In combination with the results from CD spectroscopy, the data suggested a correlation between the helix content and *G′* of the mixtures.

### Chemical cross-linking kinetics of modified gelatin derivatives

**Figure 4:**
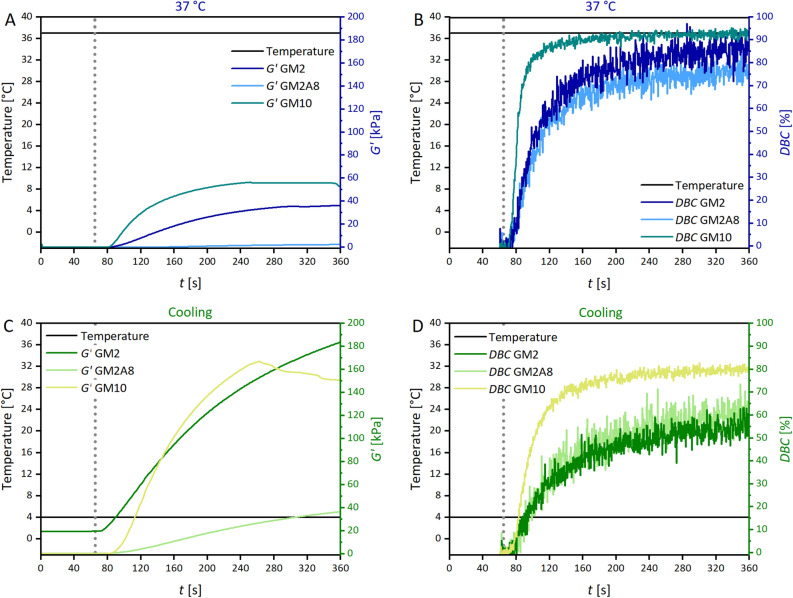
Real time-near infrared spectroscopy-photorheology data of GM2, GM2A8 and GM10 solutions. The storage moduli (*G′*) and double bond conversions (*DBC*) measured for chemical cross-linking at 37 °C (classical method) are shown in (**A**, **B**). *G′* and *DBC* measured during the sequential cross-linking procedure (cooling) are shown in (**C**, **D**), respectively. Note that the data in (**C**, **D**) were obtained directly after physical cross-linking shown in Fig. 3 using the same samples. The time axes in this figure therefore refer to the chemical cross-linking period only. Sequential cross-linking resulted in higher *G′* after chemical cross-linking and lower *DBCs* for all three gelatin derivatives. The grey dotted line indicates the start of UV-initiated cross-linking. The figure was created with Origin 2019b (https://www.originlab.com/2019b).

In the next step, the chemical cross-linking kinetics depending on the chemical gelatin modification as well as the cross-linking protocol were studied. For this purpose, we used real time-near infrared (RT-NIR) spectroscopy hyphenated with photorheology. This allows the simultaneous measuring of time-dependent changes in the storage modulus *G′* and in the double bond conversion *DBC* when using the classical photo-cross-linking method at 37 °C or sequential cross-linking including an initial cooling step. The results of time-dependent *G′* and *DBC* measurements are shown in Fig. 4. The parameters describing the initial increase of *G′* (*k*_*g,c*_) as well as the initial increase of *DBC* (*k*_*DBC*_) were calculated with the *G′*(*t*) and *DBC*(*t*) curves, respectively (Table 3), similarly to how it was described above for *k*_*g,p*_. Additionally, extracted reaction rates of double bond conversion are shown in Figure S5. The baseline for calculation of *k*_*DBC*_ was set to zero for cross-linking at 37 °C, or to the final *G′* of the physical gel for sequential cross-linking procedure (20 min at 21 °C and 40 min at 4 °C). Furthermore, the chemical gelation delay time (*t*_*d,c*_), measured from the start of UV-irradiation, and the final *DBCs* in mmol g^−1^ are given in the Supporting Information (Table S1 for the classical method, Table S2 for sequential cross-linking).

**Table 3:** Parameters describing the photo-induced chemical cross-linking of gelatin derivative solutions with the classical method at 37 °C or with the sequential cross-linking method: initial increases *k*_*g,c*_ and *k*_*DBC*_ of storage modulus *G′* and double bond conversion *DBC*, respectively, chemical gelation delay time *t*_*d,c*_ upon UV irradiation, final *G′*, and final *DBC*.

	Chemical Cross-Linking									
	Classical method (measured at 37 °C)					Sequential cross-linking (measured at 4 °C)				
	*k*_*g,c*_ (Pa s^−1^)	*k*_*DBC*_ (% s^−1^)	*k*_*DBC*_ (µmol g^−1^ s^−1^)	*G′* (kPa)	*DBC* (%)	*k*_*g,c*_ (Pa s^−1^)	*k*_*DBC*_ (% s^−1^)	*k*_*DBC*_ (µmol g^−1^ s^−1^)	*G′* (kPa)	*DBC* (%)
GM2	273 ± 14	1.9 ± 0.4	5.4 ± 1.1	36.0 ± 1.9	86.2 ± 1.2	926 ± 28	0.7 ± 0.1	1.9 ± 0.3	180.9 ± 3.3	58.1 ± 2.1
GM2A8	15 ± 1	1.7 ± 0.2	5.9 ± 0.8	2.3 ± 0.2	75.6 ± 1.2	173 ± 9	1.0 ± 0.3	3.5 ± 1.0	35.6 ± 1.9	62.1 ± 6.3
GM10	887 ± 24	5.4 ± 0.1	51.2 ± 1.3	55.6 ± 4.4	93.1 ± 0.1	1770 ± 236	2.2 ± 0.1	20.7 ± 0.8	165.1 ± 15.0	80.8 ± 0.8

The data given in % are relative to the degree of methacryloylation (DM) of the respective gelatin derivative.

The *DBC* increased rapidly for all derivatives and cross-linking conditions directly after starting UV irradiation, *G′* increased slightly delayed, compare Fig. 4A and Fig. 4B as well as Fig. 4C and Fig. 4D, respectively. At 37 °C, the *DBC* rates *k*_*DBC*_ were indistinguishable for GM2 and GM2A8 (*p* > 0.05), while *k*_*DBC*_ of GM10 was roughly 9.5 times as high when expressed as amount of converted double bonds per second. The *k*_*DBC*_ was generally lower for sequential cross-linking than for the classical method, but relative differences between the derivatives were similar in both cross-linking conditions. The results showed that both, increasing DM as well as elevated temperature, accelerated the absolute *DBC* kinetics (Figure S5 and Figure S6). These findings were expected and gave the following order *DBC* kinetics: GM10 > GM2A8 > GM2, following the order of DM of the derivatives.

However, interestingly there was no clear correlation between *k*_*DBC*_ and *k*_*g,c*_ of *G′* (Figure S6), especially because of exceptionally low *k*_*g,c*_ of GM2A8. At both cross-linking conditions, *k*_*g,c*_ decreased in the order of GM10 > GM2 ≫ GM2A8 and the resulting *G′* were significantly higher for all gelatin derivatives in the sequential cross-linking protocol when UV irradiation was carried out at 4 °C (*p* < 0.001). A similar observation was already described by van Hoorick et al*.*, who performed time-dependent photorheology measurements on a highly modified gelatin derivative with a total DM of 0.99 mmol g^−1^ and a GM with a lower DM of 0.37 mmol g^−1^ ^9^. They also followed a sequential cross-linking protocol (5 °C for 10 min prior to chemical cross-linking), but did not quantify the effect of the cooling step on the *DBC* kinetics. These results showed that the time-dependent evolution of mechanical properties of all GM(A) hydrogels was strongly influenced by the thermal history of the gelatin derivative solution.

The data on *G′* and the *DBC* in our present study now allow the conclusion that *DBC* causes an increase of *G′*, whether a physical gel was initially formed or not. The conversion rate *k*_*DBC*_ was reduced at 4 °C compared to 37 °C, also independently from the physical state of the solution. The observed strengthening effect of sequential cross-linking (i.e. elevated *G′* compared to gels that were photo-cross-linked at 37 °C) occurred for all gelatin derivatives no matter whether with rather strong (GM2), or with only weak (GM2A8), or without prior physical gelation (GM10). Particular interesting seems the fact, that GM10 hydrogels showed elevated *G′* if cross-linked after cooling at 4 °C instead of 37 °C (*p* < 0.001), although no physical gel formation occurred and the *DBC* at 4 °C was obviously lower than 37 °C (*p* < 0.001).

Given that *G′* increases with an increasing density of elastically active chains, the sequential cross-linking protocol seems to enhance the number of elastically active chains formed per converted acrylic group during UV irradiation, and it is conceivable that this is connected to diverse physical interactions and conformation changes during the cooling protocol described above.

### Correlation between stiffness and double bond conversion

**Figure 5:**
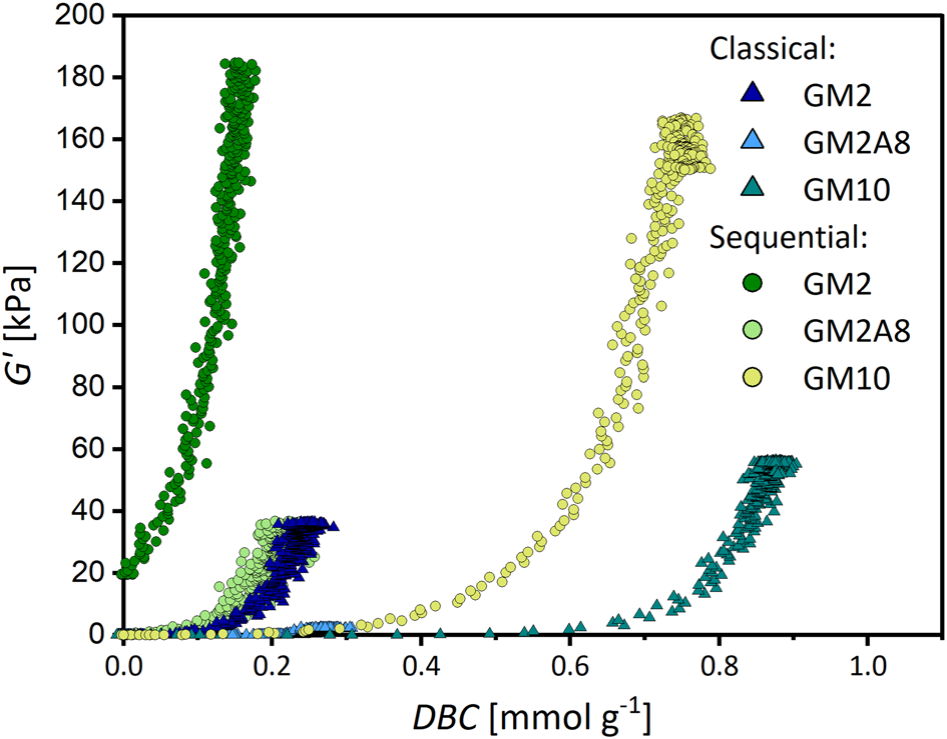
Increase of storage modulus *G′* with the double bond conversion (*DBC*) for the three gelatin derivatives GM2, GM2A8, and GM10 using the classical or the sequential cross-linking procedure, respectively. Note that the light blue triangles representing the data obtained for GM2A8 with the classical cross-linking procedure are visible close to the abscissa at *DBC*s below 0.3 mmol g^−1^. The figure was created with Origin 2019b (https://www.originlab.com/2019b).

The data on the physical interactions and chemical cross-linking of the gelatin derivatives, described in the previous sections, indicated a complex interplay between physical and chemical cross-links affecting the stiffness of the hydrogels. To further understand the correlation between the hydrogel stiffness given by *G′* and the *DBC*, the time-dependent data on *G′* and *DBC* from Fig. 4 were combined in Fig. 5. The values of final *G′* and final *DBC* relative to the respective DM are collected in Table 3. These results showed that the cross-linking of GM(A) is much more complex than usually assumed. The stiffness of GM(A) hydrogels can be adjusted by the DM, as it is often stated^3–5^, but a higher DM does not necessarily lead to stiffer hydrogels.

The *DBC* correlated in a positive sense with the number of methacryloyl functions (DM) present in the respective GM(A) derivative (GM2 ~ GM2A8 < GM10). Nevertheless, there was no systematic correlation between final *DBC* and final *G′*: as expected, *G′* of each derivative was much higher if chemical cross-linking was performed after cooling compared with chemical cross-linking at 37 °C. However, the final *DBC* of each derivative was lower for chemical cross-linking after cooling compared with chemical cross-linking at 37 °C. This was unexpected.

As Fig. 5 shows, in all cases, a non-linear increase of *G′* with increasing *DBC* was found. The impact of *DBC* on *G′* was less in GM2A8 and GM10 hydrogel precursor solutions than in GM2 hydrogel precursor solutions. Furthermore, differences in the effect of *DBC* on *G′* were observed between the two investigated cross-linking procedures and are described in the following.

For chemical cross-linking at 37 °C, *DBC* data for GM2A8 and GM10 were very similar up to the final *DBC* of GM2A8 (0.272 mmol g^−1^) and only rather low *G′* values were found. At higher *DBC* of GM10, *G′* starts to increase stronger at *DBC* of roughly > 0.6 mmol g^−1^, reaching its final value at 55.6 kPa with a *DBC* of 93.1%. For GM2, an increase of *G′* at much smaller *DBC*s (~ 0.1 mmol g^−1^) was found. In all three GM(A) hydrogels, the majority of double bonds were converted during UV irradiation. However, it was interesting that GM2 performed different from GM10 and especially from GM2A8 also at 37 °C although gel formation or triple helix formation was not detected for any of the derivatives.

Concerning the *DBC* in GM hydrogels, only very limited data existed so far^16,17,38,39^. Unfortunately, none of these studies gave any details about temperature control during curing, which would be crucial in the light of the findings reported here. All investigations used hydrogel precursor solutions out of GMs with a DM between 0.23 and 0.33 mmol g^−1^, which is similar to the DM of GM2 investigated in this study, containing the photoinitiator Irgacure 2959. However, in these studies different *DBCs* varying from 0.09 mmol g^−1^ up to 0.29 mmol g^−1^ (40–95%) were reported. Summarizing, a wide range of final *DBC*s was found in different studies and the reason is unclear so far. It is obvious that due to the multitude of experimental parameters (GM concentration, DM of GM, photoinitiator, photoinitiator concentration, solvent, UV irradiation time, UV lamp emission spectrum, sample thickness, thermal history, etc.) the comparison of datasets from different studies is difficult, especially if not all relevant experimental parameters were described in detail. Therefore, future studies should investigate the impact of experimental parameter on the *DBC.* Thereby, GM hydrogels without remaining reactive C=C-double bonds could be established, which are of special interest for application as biomaterials.

For chemical cross-linking at 4 °C (sequential protocol), effects of the *DBC* on *G′* were observed immediately after starting the chemical cross-linking at 0 mmol g^−1^ *DBC* for GM2, at 0.1 mmol g^−1^ *DBC* for GM2A8, and at 0.3 mmol g^−1^ *DBC* for GM10. This means that the contribution of the formed chemical cross-links to *G′* was much more effective for all derivatives if the solutions were cooled. However, it was unexpected that GM10 hydrogel precursor solutions performed as GM2 and GM2A8 solutions, since there was gel-formation and triple helix formation detected upon cooling for GM2 and GM2A8, but not for GM10. Thus, for GM10, right-handed single helices as postulated from the CD spectra could be the reason for the elevated effect of *DBC* on *G′* as well as the random polypeptide chain conformation, which was increased in all GM(A) solutions due to cooling.

Concerning the *DBC* at which *G′* starts to increase, the physical interactions and conformation changes caused by the cooling protocol apparently shift the *G′*-*DBC* curve to the left, see Fig. 5. This confirmed our hypothesis that *G′* of hydrogels is not solely dependent on the degree of chemical cross-linking^3,6^, and chemical cross-links might support physical cross-links stiffening sequentially cross-linked hydrogels^6,9,40,41^. However, the general shape of all *G′*-*DBC* curves were quite similar (Fig. 5). This suggests that it is irrelevant if a cross-link was based on physical or chemical interactions and that only the cross-link density itself determines the modulus, in accordance with the theory of rubber elasticity. The data suggest that an acrylic group converted in a network with already a rather high concentration of cross-links leads to larger increase of elastically active chains than in a network with a lower concentration of cross-links. Therefore, *G′* of sequentially cross-linked GM2 increased as soon as UV irradiation was started because it contained the highest density of physical cross-links (Table 1, Fig. 1), whereas *G′* of all other gelatin derivatives went through an induction period determined by the starting concentration of physical cross-links and interactions.

The lower final *DBC* and *k*_*DBC*_ in sequentially cross-linked GM(A) hydrogels refuted the hypothesis that sequential cross-linking increases the efficiency of chemical cross-linking, established in earlier studies^6,9,14,40,41^. A possible explanation could be that methacryloyl groups lose mobility and get inaccessible by gelation and network organization due to the cooling procedure, as mentioned by Park et al*.*^14^. The increase of *G′* at lower *DBCs* after cooling indicate that such obstacles as the physical gelation hinder chemical cross-linking already in an earlier phase of the chemical cross-linking reaction, ultimately limiting the achievable *DBCs*. The fact that the *DBC* of GM2 was most affected by the restrictions caused by physical gelation is consistent with this explanation, as also indicated by the higher *DBC* of GM2A8 compared to GM2.

Interestingly, our observation of smaller *DBC*s after sequential cross-linking seems to contradict findings by Rizwan et al*.*^8^. They reported a decrease in unreacted double bonds in 30% GM hydrogels prepared from a GM with a DM of 0.31 mmol g^−1^ due to their sequential hydrogel preparation procedure (4 °C for 1 h) compared to cross-linking at 37 °C, measured by qualitative Fourier transform infrared spectroscopy (FTIR). Therefore, it cannot be ruled out that differences in experimental conditions have strong impact on the development of material properties. This is known from unmodified gelatin as well and strict compliance to standard protocols is required if standard parameters like viscosity, gel strength etc. are determined for commercial products.

Concerning the final *G′*, the observed effects in this study are in agreement with previous reports^6–9,11^, and we confirmed the stiffening effect of sequential cross-linking even for GM hydrogel precursor solutions, which do not form physical networks^6,9^. The seemingly reasonable assumption that physical interactions do not play any role when cross-linking above the physical gel melting temperature of gelatin derivatives, e.g*.* at 37°, was disproved, especially by the remarkable difference in stiffness of GM2 and GM2A8 hydrogels at comparable *DBCs* when using the classical cross-linking procedure: also at 37 °C, differences in physical interactions significantly alter hydrogel properties.

Summarizing, with the sequential cross-linking method, three main differences were observed compared to the classical method for a given gelatin derivative: (1) The final *DBC* was generally lower, (2) the final *G′* was generally higher, and (3) *G′* increased at lower *DBC* values. Since we measured in this study lower *DBCs* during sequential cross-linking than during chemical cross-linking, we concluded that the physical interactions and conformation changes induced by the cooling protocol (see Figs. 1, 2) were effective for stiffening all sequentially cross-linked hydrogels. However, the individual contributions for each GM derivative seem to depend on the DM and total degree of modification. The data in this study were obtained without including GMs with lower DMs, and there might be an optimum DM with an optimum sequential cross-linking procedure leading to the maximum hydrogel stiffness. Lower DMs, however, cause too shallow absorption bands in the NIR spectra and would be difficult to analyse. Further insight will be necessary to find out if additional effects have to be considered to be responsible for the observed correlations between *DBC* and *G′*.

In any case, it cannot be emphasized enough that the experimental conditions must be controlled as precise as possible and the conditions applied need to be described in detail to reproduce experiments. We have to remark that the final *G′* values in this study were measured for hydrogels in their relaxed state and not in their swollen state. Therefore, a direct comparison with hydrogels swollen to equilibrium is not possible^9^.

## Conclusions

The stiffness of gelatin methacryloyl (GM) hydrogels was increased dramatically by sequential cross-linking including an initial cooling step. We refuted the previously established hypothesis that a more effective double bond conversion (*DBC*) leads to this stiffening: cooling before chemical cross-linking in fact caused lower *DBCs* in all GM hydrogels. Triple helix formation was detected for GM2 and GM2A8, and single helices with cis-peptide bonds (right-handed single helices) were detected in GM10 and GM2A8 solutions upon cooling. Therefore, we propose that these physical interactions, which lead to ordered conformational structures, caused the steep increase of the stiffness with low *DBC*s*,* possibly resulting in higher total numbers of physical and chemical cross-links. Consequently, the combination of conformational structures based on physical interactions and chemical cross-links is necessary to obtain gelatin derivative hydrogels with high structural strength. These findings are important e.g*.* in applications of GM hydrogels in cartilage tissue engineering where strong hydrogels are desired while keeping the degree of modification at a low level.

## Supplementary Information

Supplementary Information
