# Supplementary material for: Differentiation of physical and chemical cross-linking in gelatin methacryloyl hydrogels
Source: Sci Rep. 2021 Feb 5;11:3256. doi: 10.1038/s41598-021-82393-z (PMC7864981; doi:10.1038/s41598-021-82393-z)
Supplement: Supplementary file 1 — Supplementary Information [file 41598_2021_82393_MOESM1_ESM.pdf]

Supplementary Information

Differentiation of Physical and Chemical Cross-Linking in Gelatin Methacryloyl Hydrogels

Lisa Rebers[1,+], Raffael Reichsöllner[2,+], Sophia Regett[1], Günter E. M. Tovar[1,3,*], Kirsten Borchers[1,3], Stefan Baudis[2] and Alexander Southan[1,*]

[1] Institute of Interfacial Process Engineering and Plasma Technology, University of Stuttgart, Stuttgart, Germany.

[2] Christian Doppler Laboratory for Advanced Polymers for Biomaterials and 3D Printing, Institute of Applied Synthetic Chemistry, TU Wien, Vienna, Austria.

[3] Fraunhofer Institute for Interfacial Engineering and Biotechnology, Stuttgart, Germany.

* alexander.southan@igvp.uni-stuttgart.de, guenter.tovar@igvp.uni-stuttgart.de

[+] These authors contributed equally.

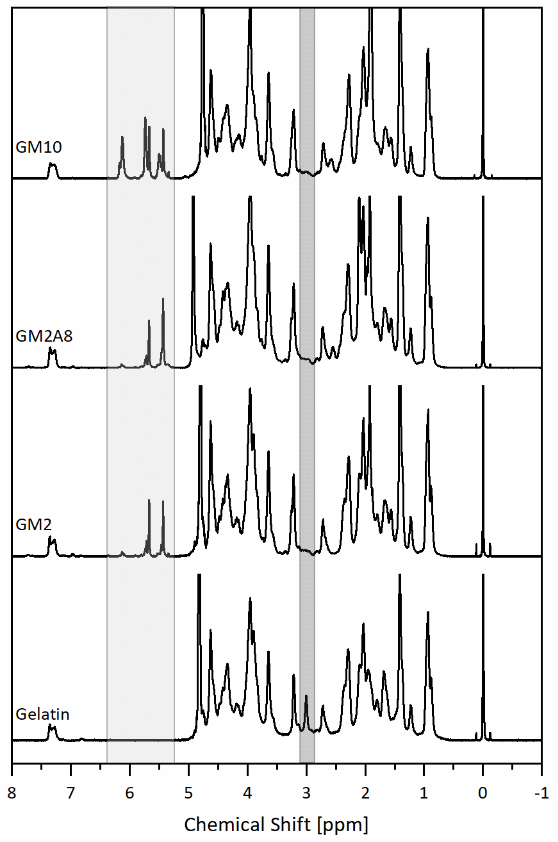

Figure S1: ^{1}H-NMR spectra of gelatin used for methacryloylation (GM) (and acetylation (GMA)) and its derivatives. Unmodified lysine groups, only present in the spectrum of the unmodified gelatin, were highlighted in dark grey, acrylic protons of methacryloyl groups in light grey. The figure was created with Origin 2019b (https://www.originlab.com/2019b).

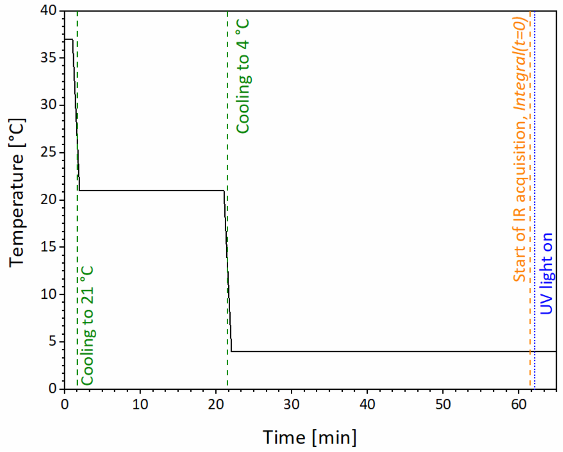

Figure S2: Utilized temperature profile for physical gelation prior to chemical cross-linking. The 37 °C warm GM-solutions were cooled for 20 min to 21 °C followed by cooling to 4 °C 40 min (green dotted lines). Afterwards, infrared spectroscopy (IR) acquisition was started (orange dotted line) and the UV light was turned on 5 s later (blue dotted line). The figure was created with Origin 2019b (https://www.originlab.com/2019b).

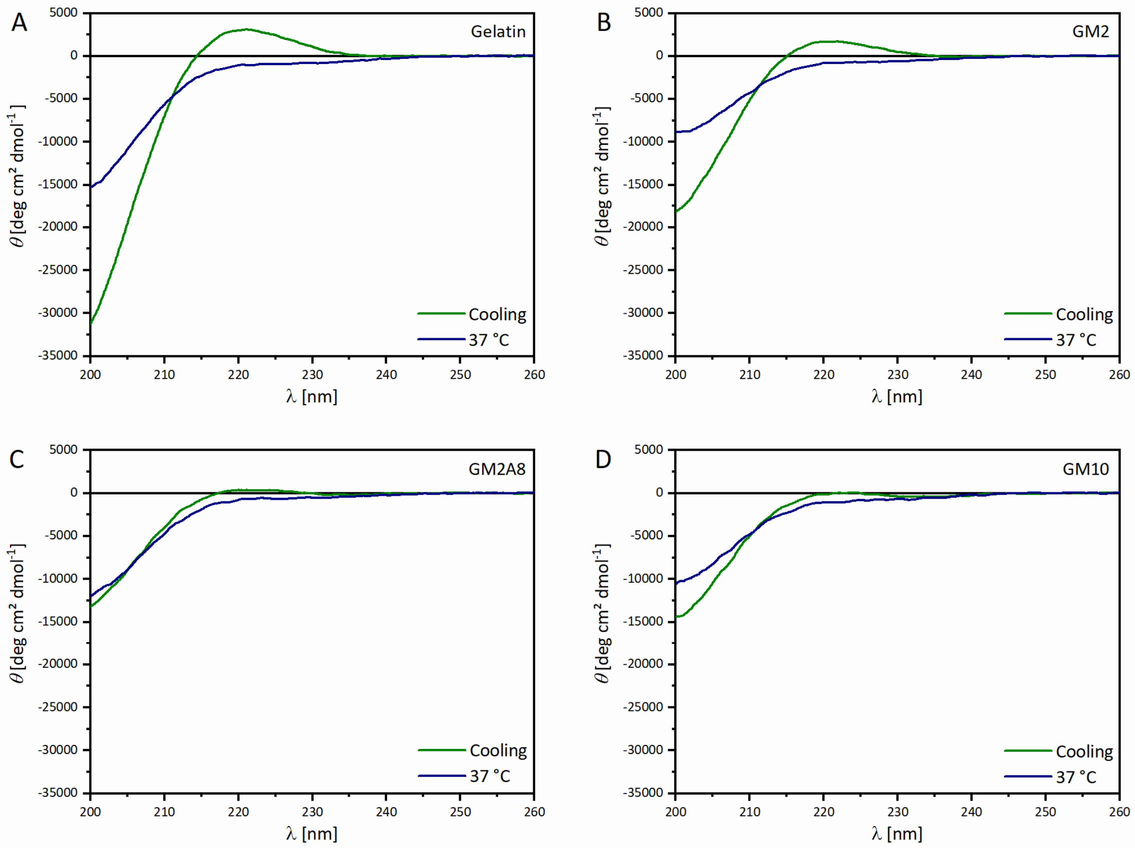

Figure S3: Circular dichroism (CD) spectra of gelatin (**A**), GM2 (**B**), GM2A8 (**C**) and GM10 (**D**). CD spectra were recorded at 37 °C or after cooling procedure (37 °C to 21 °C for 20 min followed by cooling to 4 °C for 40 min). The figure was created with Origin 2019b (https://www.originlab.com/2019b).

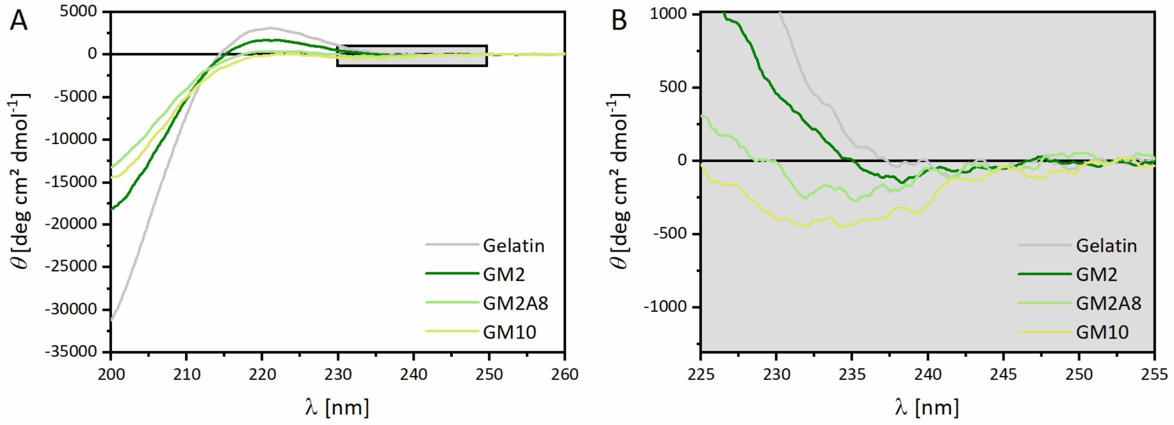

Figure S4: Circular dichroism (CD) spectra of gelatin (derivatives) after cooling procedure (**A**) and a zoom-in of these CD spectra between 225-255 nm (**B**). The figure was created with Origin 2019b (https://www.originlab.com/2019b).

Table S1: Chemical gelation delay time ($t_{d,c}$) and final double bond conversion (*DBC*) in mmol g^{-1} of GM2, GM2A8 and GM10 cross-linked with the classical method at 37 °C.

	$t_{d,c}$ [s]	DBC [mmol g^{-1}]
**GM2**	25.0±2.7	0.250±0.003
**GM2A8**	72.9±1.5	0.272±0.004
**GM10**	18.3±0.6	0.885±0.001

Table S2: Chemical gelation delay time ($t_{d,c}$) and final double bond conversion (*DBC*) in mmol g^{-1} of GM2, GM2A8 and GM10 cross-linked with sequential cross-linking protocol (starting at 37 °C, cooling to 21 °C for 20 min followed by cooling to 4 °C for 40 min).

	$t_{d,c}$ [s]	DBC [mmol g^{-1}]
**GM2**	12.2±0.3	0.168±0.006
**GM2A8**	34.3±1.5	0.224±0.023
**GM10**	31.2±1.7	0.768±0.008

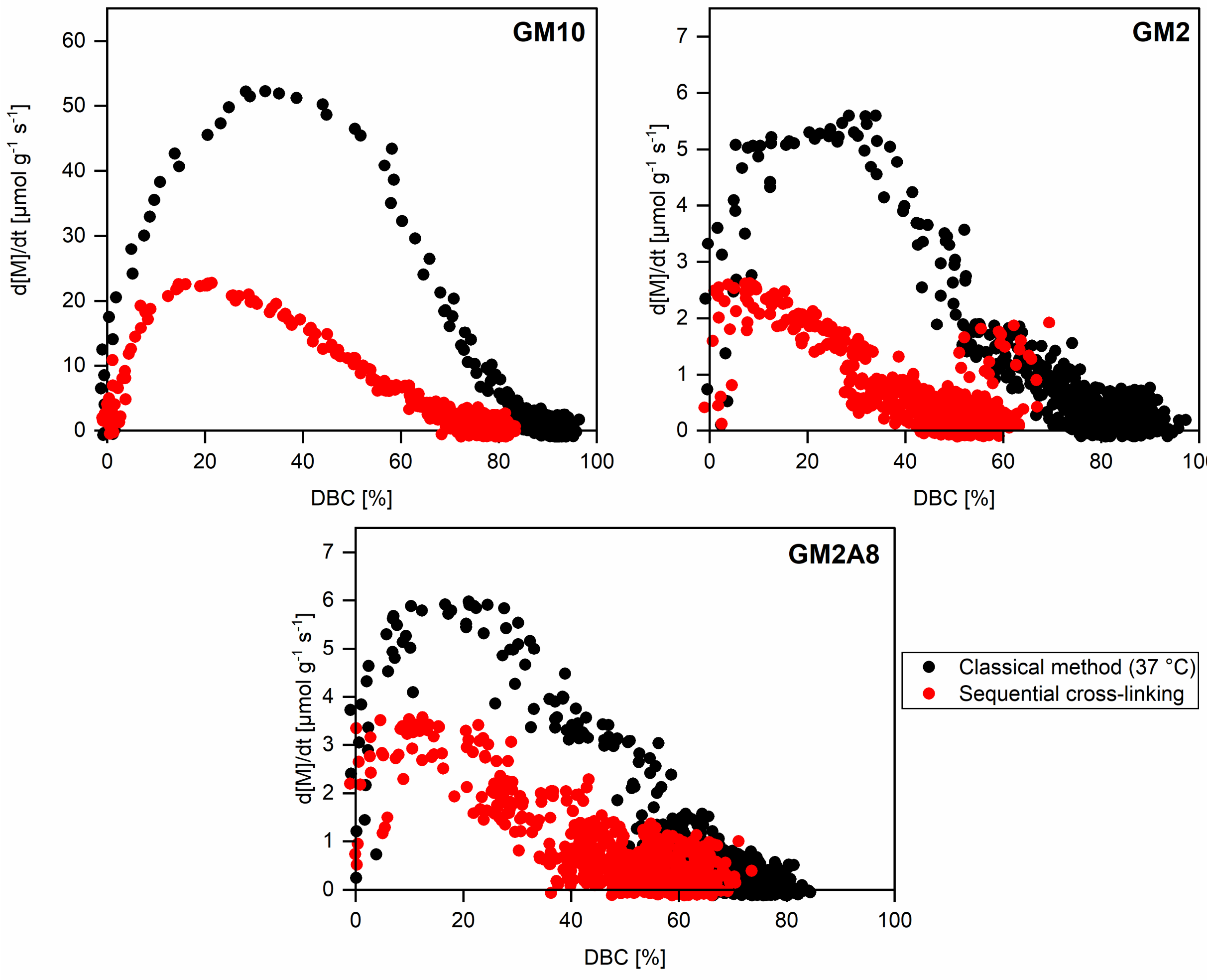

Figure S5 Reaction rate of double bond conversion against double bond conversion for GM10, GM2, and GM2A8. The figure was created with Origin 2019b (https://www.originlab.com/2019b).

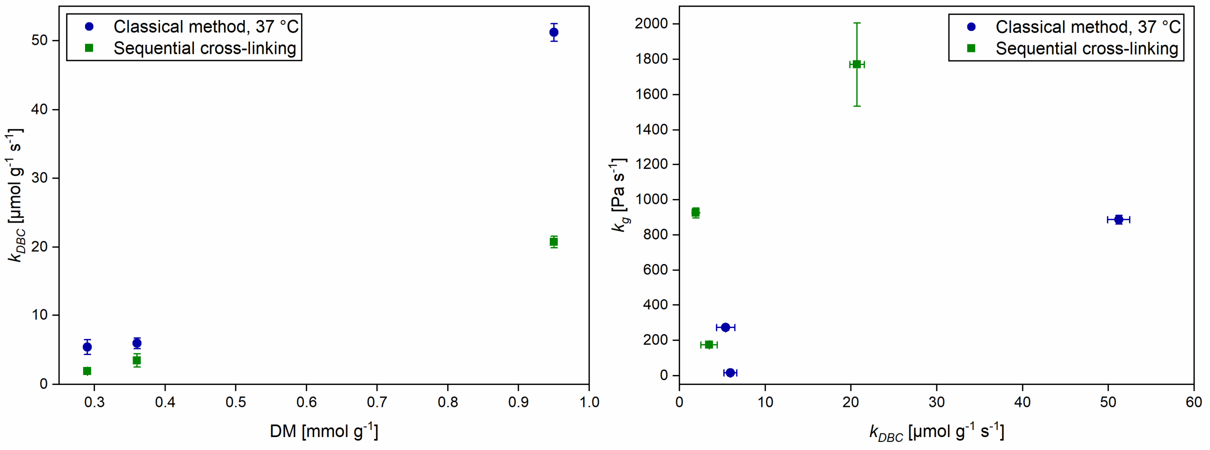

Figure S6: Correlations between k_{DBC} and DM (left) as well as k_g and k_{DBC} (right). The figure was created with Origin 2019b (https://www.originlab.com/2019b).
